# Brief Report: The Rise of Online Betting in Ontario

**DOI:** 10.1007/s10899-023-10268-1

**Published:** 2023-12-13

**Authors:** Nigel E. Turner, Lindsay Sinclair, Flora I. Matheson

**Affiliations:** 1 Institute for Mental Health Policy Research, and Campbell Family Mental Health Research Institute, Centre for Addiction and Mental Health, 33 Ursula Franklin Street, Toronto, ON M5S 2S1 Canada; 2 Dalla Lana School of Public Health, University of Toronto, 155 College Street, 6th Floor, Toronto, ON M5T 3M7 Canada; 3 Problem Gambling and Technology Use Treatment Service, Centre for Addiction and Mental Health, Toronto, ON Canada; 4 Department of Occupational Science and Occupational Therapy, University of Toronto, Toronto, ON Canada; 5 MAP Centre for Urban Health Solutions, St. Michael’s Hospital, Toronto, ON Canada; 6 Dalla Lana School of Public Health, University of Toronto, Toronto, ON Canada

**Keywords:** Helpline data, Crisis calls, Sports gambling, Online gambling, Time series analysis

## Abstract

In this paper we examine the nature of calls for the Ontario Problem Gambling Helpline from June 2021 to Jan 2023 to determine if the increased marketing of online and sports gambling has changed the nature of calls to the helpline. An interrupted time series model comparing the monthly calls before and after the expansion of online gambling and advertising (April 2023), found a significant effect. Calls related to the other games examined did not have a significant interrupted time series effect of from the expansion and advertising of online gambling. The results of this analysis clearly indicate an association between the expansion of legalized gambling and gambling advertising with the number of people who call the helpline for problem related to online gambling.

Sports betting and online gambling in Canada has changed rapidly since 2020. In 2021, the government of Canada legalized single event sports betting, signed into law on August 27, 2021 (Evans, 2021). In anticipation of this legal change the Ontario Problem Gambling Helpline (OPGHL) started recording mentions of online gambling their administrative database in June 2021. In April 2022, the Ontario government expanded the legal framework for gambling in Ontario legalizing commercial companies to offer online gambling and single event sports gambling and permitting advertising of internet gambling in Ontario (Pesaruk, 2022). This allowed for a massive increase in advertisements for sports and online gambling (reference?). As a result, people in Ontario (and across Canada) have been inundated with advertisements for sports betting and online casino gambling, often using celebrities (Pesaruk, 2022). In this paper we examine the nature of calls for the Ontario Problem Gambling Helpline from June 2021 to Jan 2023 to determine if the increased marketing of online and sports gambling has changed the nature of calls to the helpline.

Traditionally electronic gambling machines (EGMs) account for the majority of revenue for the gambling industry (Turner, 2011). In addition, numerous studies have reported that EGM games in casino and other venues account for the majority of gambling problems (Binde, 2011; Delfabbro, et al., 2020; Dorion & Nicki, 2001; Turner, 2011; Williams, et al., 2021). In a previous study Turner et al. (2020; 2022), used helpline calls to examine how the closing of casinos during the Covid-19 pandemic resulted in a sudden decrease in helpline related calls and a gradual shift in the demographics of callers to the helpline. The reduction in calls was particularly large for EGM-related calls. They found that after this initial reduction, helpline calls began to return to pre-pandemic levels even though casinos remained closed. In addition, they reported a shift in the types of games played, with relatively less slot machine play and relatively more sports and card game play. They also found that more younger people and males were calling the helpline, which they suggested is consistent with more online gambling. (Turner, et al., 2022). During this time period, online was only legally available through the Ontario Lottery and Gaming web site. However, online gambling and sports betting was easily available from online gambling providers anywhere in Canada (Mergulhao, 2020). No effort had been made to limit access to this grey market for gambling online (Mergulhao, 2020). Data from around the world also suggests that Covid-19 lead to a shift towards more online gambling (Andersson et al., 2022; Brown & Hickman, 2020; Håkansson et al., 2020; ResearchAndMarkets.com, 2020; The Economist, 2020; Saeed et al., 2023). Previous studies have shown that the marketing of gambling increases gambling (Killick & Griffiths, 2022; McGrane et al., 2023; Wardle, et al., 2022).

The OPGHL began recording mentions of online gambling in June 2021. This coincided with the announcement of the pending legalization of single event sports betting in Canada. The Ontario government took several months to put into place a new regulatory framework for offering single event sport betting over the internet. This new framework consisted of licencing a number of online gambling providers to offer online gambling and sports gambling over the internet. It was hypothesized that calls related to online gambling would increase after legalization of commercial online gambling in April 2022, and subsequence increase in legal advertising for online gambling and sports gambling.

## Method

### Data

Gambling helpline data for calls from June 2021 to the end of January 2023 were provided by ConnexOntario for analysis. The data were obtained by ConnexOntario. The calls are confidential, so no information about the callers was disclosed. The information is aggregated by month (i.e., not individual level data), anonymous, and publicly available. As such, no ethical review is required to analyse or publish the data.

The Helpline is available toll free by telephone and on the internet at ConnexOntario. The helpline number appears on all lottery tickets, is posted in the responsible gambling centres located in casinos, on slot machines, advertising for the gambling industry in Ontario, and on all legal websites related to gambling in Ontario.

### Measures

According to Turner, et al., (2022) only 57% of people who call into the helpline report the type of gambling they participate in. A total of 2677 calls identified the game they were having problems with (69.0% male). Table 1 provides the distribution of online related calls and all other calls from people calling for help with gambling problems. Of those calls, the largest number were from people aged 25 to 34 (31%) and aged 35 to 44 (21.3%).

**Table 1:** Demographics of people who call regarding online and all other games

	All other games (%)	Online (%)	Total (%)
*Gender*			
Female	27.6%	22.7%	26.6%
Male	67.7%	74.3%	69.0%
Non-binary/Gender fluid	0.2%	0.0%	0.1%
Not identified	4.6%	3.1%	4.3%
*Age*			
19 or under	7.4%	3.9%	6.6%
20–24	11.0%	15.0%	11.8%
25–34	29.6%	37.6%	31.3%
35–44	21.0%	22.3%	21.3%
45–54	16.8%	10.3%	15.5%
55–64	8.7%	7.2%	8.4%
65+	5.5%	3.6%	5.1%

“All other games” include people who did not identify the game they had a problem with which was about 43% of the people who called the helpline

ConnexOntario collected information on 13 types of gambling which we grouped into six categories: (1) Electronic Gaming Machines (EGMs) such as slot machines, and video lottery terminals, (2) lotteries such as scratch tickets and large draws, (3) table games including poker and blackjack, (4) sports betting, (5) online gambling, and (6) other games including stock market, racing, and bingo.

Because of the large impact of COVID-19 on helpline calls (Turner, et al., 2022) we also re-examined the data reported in Turner et al., (2022) to help understand the context of the current analysis; namely how the numbers in the current analysis compare to calls before and during the pandemic.

The two main analyses of interest was the number of calls related to different types of gambling and the effect of the legalization before and after the expansion of advertising for online gambling in April 2022. The change in legislation changes will be examined using an interrupted time series analysis (ITSA).

### Statistical analysis

First, we examined the changes in the number of calls for each type of gambling and the changes in the percentage of calls of each type of gambling. We then examined the call data using interrupted time series analyses (ITSA) to assess the changes in call trend before and after the change in legislation in April 2022. Preliminary analysis of autocorrelations was conducted using IBM SPSS statistics version 27. The ITSA was conducted using Stata/MP 14.1 for windows. The data was first tested using the Phillips-Perron unit root test (Phillips & Perron, 1988). This test yields a significant result if the data is stationary. Because the errors followed an AR(1) process, the ITSA models were fit using the Prais method (Linden, 2015). These generalized least squares models are estimated using robust standard errors and remove the first order autocorrelation (Linden, 2015).

## Results

Of those calls that identified as either male or female (excluding non-binary and those not identified), 72.2% of the callers were male. There was no significant difference in the relative proportion of males before the legalization and regulation of commercial online gambling in Ontario, 71.5%, and after, 72.7%, Chi-square(1) = 1.09, ns. However, when we compared calls related to online gambling and all other calls for problems with gambling (excluding non-binary and not identified), calls for online gambling were significantly more likely to be males, 76.6%, than the calls related to other types of gambling 71.0%, Chi-square(1) = 14.5, *p* < 0.001.

The age range for people calling for gambling related problems did not differ significantly before or after the legalization and regulation of commercial online gambling in Ontario, Chi-square(6) = 9.2, ns. However, as shown in Table 1, a higher portion of call for online gambling were from people between 25 and 34 years old (37.6% vs. 29.6%), and from people 20 to 24 years old (15.0% vs. 11.0%) and relatively fewer calls for online gambling form people 45–54 years old (10.3% vs. 16.8%), Chi-square(6) = 83.2, *p* < 0.001. Interestingly a post hoc examination contrasting people under the aged 19 or less, were less likely to call regarding online gambling compared to other forms of gambling (3.9% vs. 7.4%), Chi-square(1) = 17.5, *P* < 0.001.

Table 2 provides a summary of game types reported by people who called the helpline during 4 times periods: (1) prior to the Covid-19 pandemic, Jan 2016 to Feb 2020, (2) during the height of the pandemic when the casinos remained closed (March 2020 to May 2021), (3) after ConnexOntario began collecting data on calls related to online gambling in anticipation that single event sports betting would be legalized, June 2021 to March 2022, (4) after the launch of licenced online and sports gambling in Ontario and the expansion of marketing of sport gambling.

**Table 2:** Monthly average calls and percentage of calls broken down by game type before the pandemic (Jan 2016 to Feb 2020), during the pandemic (March 2020 to May 2021), after ConnexOntario began collecting data on calls related to online gambling (June, 2021 to March 2022), and after the launch of expanded online gambling with heavy advertising (April, 2022 to Jan 2023)

	Time period		Online gambling	EGMs	Table games	Sports	Lotteries	Other	Total
*Calls where a game type is mentioned*									
1	Jan 2016 to Feb 2020	M	–	64.8	34.3	12.5	11.7	7.8	131.1
		SD		11.9	8.2	4.3	4.2	3.2	21.7
2	March 2020 to May 2021	M	–	15.1	14.8	9.1	8.13	3.4	50.5
		SD		8.0	4.8	4.0	2.8	2.3	15.0
3	June 2021 to March 2022	M	29.0	20.9	11.9	7.2	10.5	4.1	83.6
		SD	11.9	8.3	5.1	3.5	3.4	2.7	23.8
4	April 2022 to Jan 2023	M	92.9	35.4	24.6	16.6	8.9	5.7	184.1
		SD	24.4	7.71	6.43	3.1	2.13	4.4	31.8
*Percentage of calls*									
1	Jan 2016 to Feb 2020	M	–	49.5%	26.2%	9.5%	9.0%	5.9%	
		SD		5.3%	4.6%	2.7%	3.0%	2.1%	
2	March 2020 to May 2021	M	–	28.7%	29.9	18.3%	16.5%	6.7%	
		SD		7.3%	7.82	8.3%	5.1%	4.2%	
3	June 2021 to March 2022	M	34.2%	25.2%	14.0%	8.9%	13.0%	4.7%	
		SD	9.8%	7.4%	3.9%	4.5%	4.7%	2.4%	
4	April 2022 to Jan 2023	M	50.0%	19.4%	13.4%	9.2%	5.0%	3.1%	
		SD	6.5%	3.8%	2.6%	2.0%	1.7%	2.5%	

Time periods: (1) prior to the Covid-19 pandemic, (2) during the height of the pandemic, (3) OPGHL began recording online gambling, (4) the launch of licenced online and sports gambling in Ontario

Calls related to most forms of gambling increased from June 2021 to January 2023. For example, calls related to tables games increased from an average of 11.9 per month to 24.6 per month and EGM related calls increased from an average of 20.9 per month to 35.4 per month. Online games increased from 29.0 per month to 92.9 per month. Prior to the pandemic 49.5% of the calls were related to EGMs and other electronic gambling machines. During the pandemic EGM related calls dropped to 29.9% of calls. As shown in Table 2, EGM related calls dropped to 25.4% of the calls after the legalization of single event sports bets and to 20.1% after the introduction of expanded online and sports advertising in Ontario. Statistical analysis of the change in percentages found that the percentage of calls for online gambling significantly increased after the expanded marketing of online gambling, from 34% prior to the marketing of online gambling, to 48% after the expanded marketing of gambling, t(18) = 4.42, *p* < 0.01. In addition, the percentage of calls related to lotteries decreased from 13 to 6%, t(18) = 5.11, *p* < 0.001 and the percentage of calls related to EGMs decreased from 25.2% to 19.4%, t(18) = 2.2, *p* < 0.05 after the introduction of expanded marketing of online gambling. The percentage of other games did not change statistically.

After they began recording online gambling, online play quickly became the most frequently endorsed type of gambling. As covid restrictions were gradually removed, there was an increase in calls related to most forms of gambling (except lotteries). EGM calls increased from 15 per month during the peak of the pandemic, to 20 per month after June 2021, and finally to 35 per month from April 2022 to Jan 2023. However, 35 per month is little more than half as many calls per month compared to the 65 per month prior to the pandemic.

**Fig. 1:**
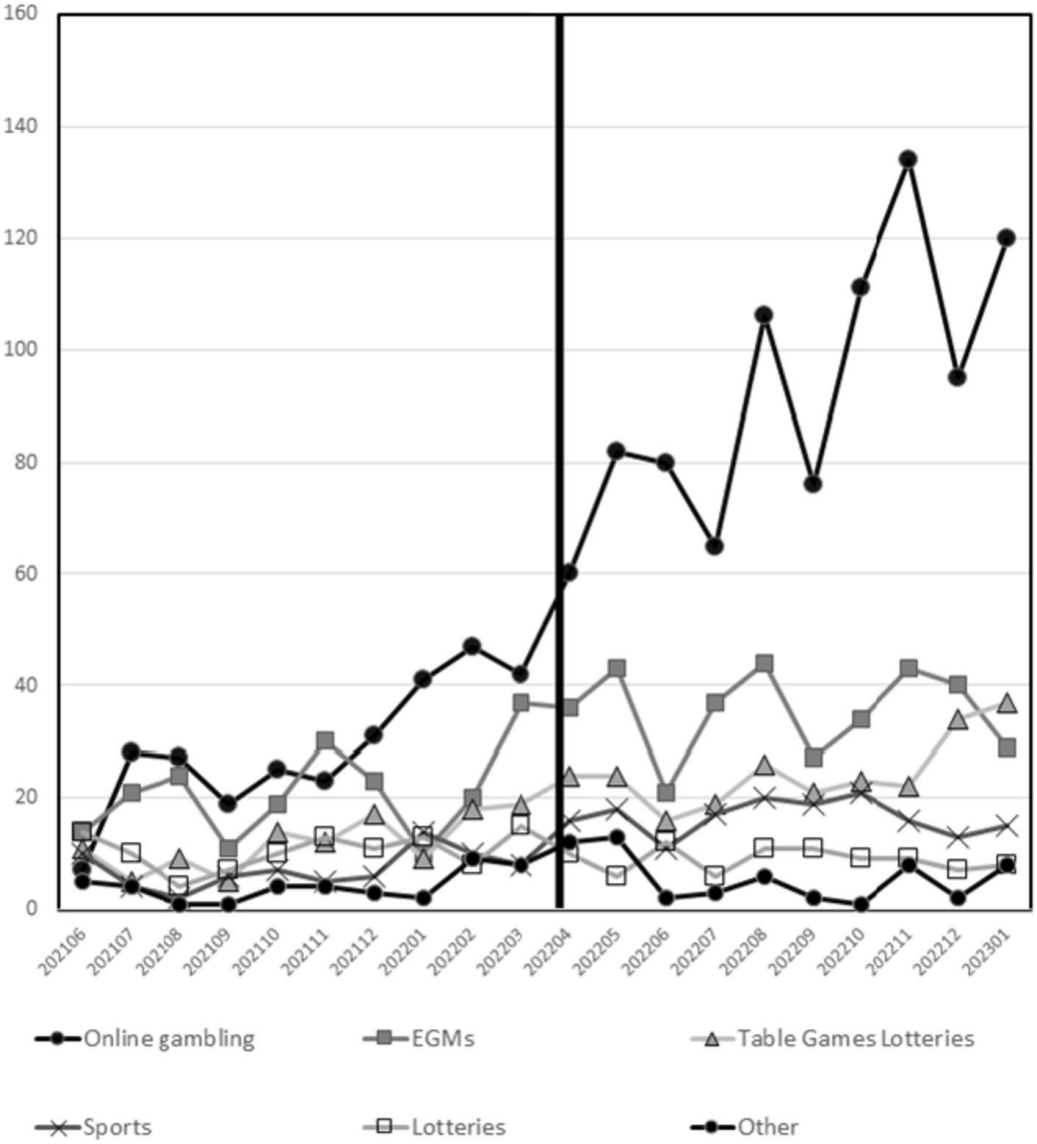
Number of calls per game type from June 2021 to January 2023. The change in legislation is indicated by the solid vertical bar, April 2022

**Fig. 2:**
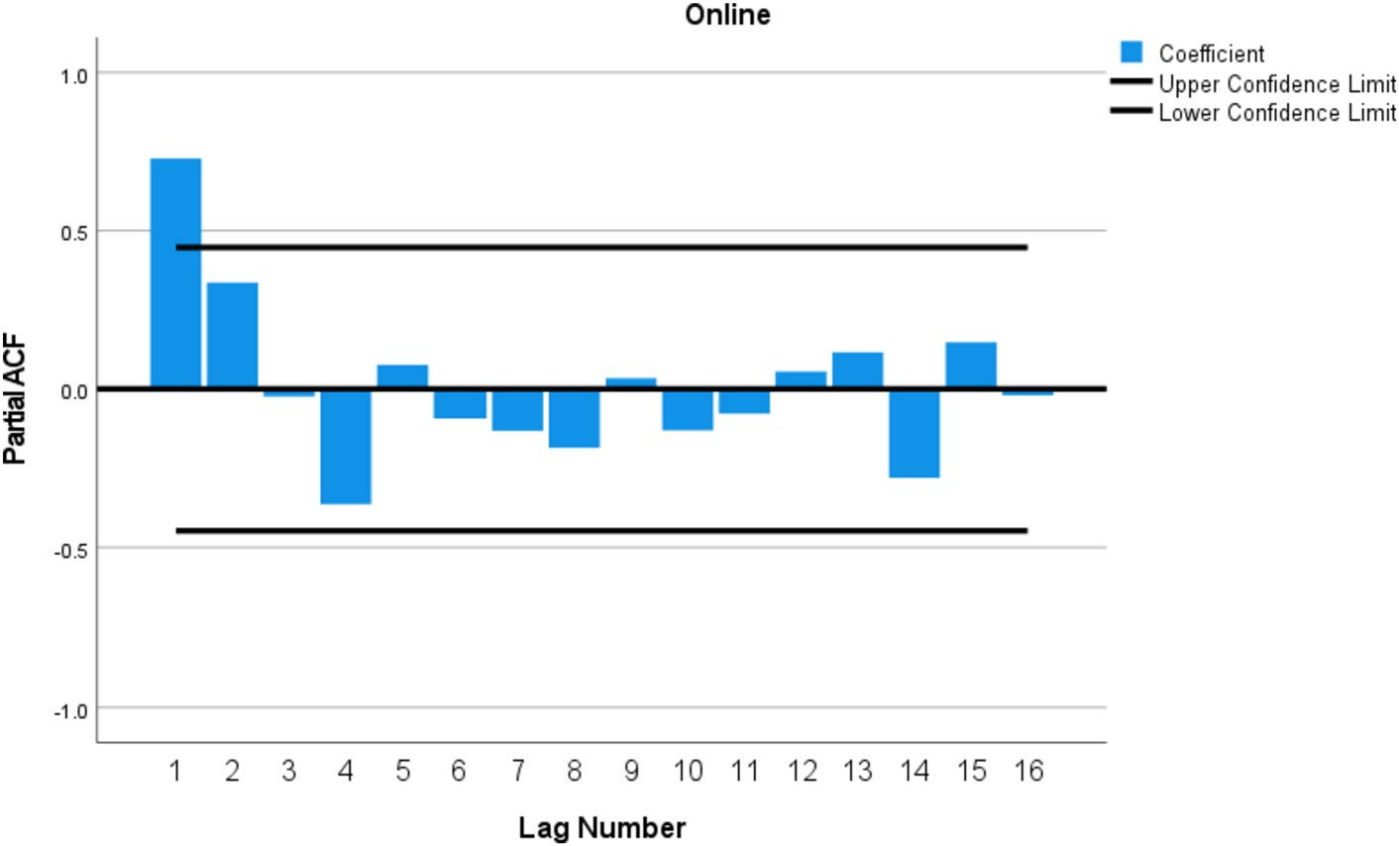
Partial autocorrelations for calls related to Online gambling

We next conducted a time series analysis of online calls to determine if the expanded advertisement and promotion, starting April, 2022, had a significant impact of the number of calls. Figure 1 is a graph illustrating how calls for the 6 types of gambling changed over time. First, we conducted an analysis of autocorrelations. As with most time series data, each data point may be correlated with the data points preceding it. As show in Fig. 2. Analysis of autocorrelation determined that only the lag 1 autocorrelation was significant.

Next, we tested if the data was stationary, using the Phillips-Perron unit root test (Phillips & Perron, 1988). This test yields a significant result if the data is stationary. A stationary data set is necessary for correct interpretation of the data. Given the strong upward trend in the data, it was not initially stationary, however after including a term for the trend, the Phillips-Perron test was significant indicating that the data was stationary, z(t) = 4.7, *p* < 0.001.

Finally, to test the interrupted time series model of the legalization of advertising and expanded gambling on calls related to online gambling, we used Prais-Winsten AR(1) regression analysis. The autocorrelations were handled using a single lag ordinary least square of residuals. The linear trend was highly significant, t = 5.7, *p* < 0.001 and the effect of legalization also had a significant additional impact of the number online calls, t = 2.2, *p* < 0.05. None of the other games had a significant interrupted time series effect of from the expansion and advertising.

## Discussion

The results of this analysis clearly indicate an association between the expansion of legalized gambling and gambling advertising with the number of people who call the helpline for problems related to online gambling. This effect was significant above and beyond the general trend of increasing calls to the helpline after the substantial drop in gambling related calls during the Covid-19 pandemic (Turner et al., 2022). It is clear from Fig. 1, that online gambling showed a particularly strong increase over this time period and that increase was greatly accelerated by the launch of the new regulatory framework that expanded the legal market for online gambling in Ontario. As noted by Turner et al., (2022) the drop in gambling related calls during the pandemic illustrates the impact of availability of gambling opportunities on gambling related problems (Storer et al, 2009; Welte et al., 2016). The current results of this study suggest that expanded gambling opportunities and the heavy marketing of online gambling lead to an increase in the number of helpline calls regarding online gambling. Unfortunately, we cannot separate the impact of the expansion of legal online gambling from the heavy marketing of online gambling because these two changes occurred at the same time.

The effect marketing of gambling is not unprecedented. Previous studies have shown that the marketing of gambling increases gambling (Killick & Griffiths, 2022; McGrane et al., 2023; Wardle, et al., 2022). In particular that the marketing of sport gambling increases unplanned gambling spending (Wardle, et al., 2022). Of particular interest is that in 2022 the UK placed limits on how sports gambling is marketed (BBC News, 2022), In particular they banned marketing of gambling during a game and in any ads directed towards people under 25.

In the past, many studies have reported that EGMs accounted for a majority of gambling related problems (Binde, 2011; Delfabbro, et al., 2020; Dorion & Nicki, 2001; Turner, 2011; Williams, et al., 2021). The results of this study suggest that EGMs may no longer be the main reason for gambling related problems. Although slot machine gambling related calls also increased during this time period, slot machine gambling did not return to the pre-pandemic level. Rather, the calls related to EGMs are currently only about half of the level of EGMs prior to the pandemic. ConnexOntario has recently began breaking online gambling down into different games (Sports, Cards, EGMs) and thus in future research we will be able to examine the type of online gambling that people are participating in and also be able to compare the rates of problems of strategic online games (cards and sports) with non-strategic online games. In addition, the data did suggest a that online gambling appeals more to males and to younger players. On a positive note, the lower rates of online gambling for people 19 and under suggests that the age restrictions on the gambling web sites are working.

## Limitations

There were several limitations in this study related to the information included in the helpline data. Another limitation is because the helpline is anonymous, one person could call several times, making it possible that the number of unique individual callers is exaggerated. However, this was also true before and after the legalization of online gambling and advertising. Previous studies have found that only a fraction of those experiencing problems with gambling end up contacting help services and do so as a last resort, even though early interventions would be more effective (see e.g., Slutske, 2006; Petry et al., 2017). Helpline calls are an indirect proxy measure of problem, and it is possible that the prevalence of actual gambling problems may not be accurately reflected in calls for problem gambling. Nonetheless, higher public awareness of helplines and their popularity as a point of first contact with professional help (Gainsbury et al., 2014; Weinstock et al., 2011; Williams et al., 2007) means that helpline data is likely the best indicator of short-term changes in gambling-related crises in the general population. Finally, the information provided by the helpline was incomplete. While most of the callers provided their age and gender, only 57% reported the specific game types played. Furthermore, during the time period under study, the games played online were not recorded. And finally, the effects of the pandemic and the change in legislation are still ongoing, and their impacts on gambling and gambling-related harms is evolving. We will continue to monitor this data to gauge the full impact of the pandemic, which will become more apparent as time passes.

## Conclusions

Although the number of calls to the OPGHL is not a direct measure of problem gambling, it can be speculated that there may be a correlation between an increase in gambling related calls to helpline and problem gambling levels in Ontario. Moreover, it is likely that the legalization and the robust advertising campaign of online gambling may be contributing to an increased level of problem gambling in Ontario. Furthermore, it can be reasoned that patrons who previously gambled at EGMs prior to pandemic may have shifted to online EGMs during the pandemic and have not returned to in-person casino games, which likely in part accounts for the decrease in slot machine gambling and increase in online gambling. Future studies are warranted to explore these hypothesis to determine if there is a connection between increased gambling related calls to the helpline and overall problem gambling incidence, and if individuals who currently play online EGMs were previous users of in-person slot machines prior to the pandemic.

## Data Availability

The data used in this study is publicly available from ConnexOntario. Go to the following web site for more information about contacting their administration https://www.connexontario.ca/contact-us.
